# The PD-1/PD-L1 Axis and Virus Infections: A Delicate Balance

**DOI:** 10.3389/fcimb.2019.00207

**Published:** 2019-06-13

**Authors:** Günther Schönrich, Martin J. Raftery

**Affiliations:** Charité – Universitätsmedizin Berlin, Corporate Member of Freie Universität Berlin, Humboldt-Universität zu Berlin, and Berlin Institute of Health, Institute of Virology, Berlin, Germany

**Keywords:** PD-1, PD-L1, PD-L2, antiviral immune responses, viral immune evasion, virus-induced immunopathogenesis, viruses

## Abstract

Programmed cell death protein (PD-1) and its ligands play a fundamental role in the evasion of tumor cells from antitumor immunity. Less well appreciated is the fact that the PD-1/PD-L1 axis also regulates antiviral immune responses and is therefore modulated by a number of viruses. Upregulation of PD-1 and its ligands PD-L1 and PD-L2 is observed during acute virus infection and after infection with persistent viruses including important human pathogens such as human immunodeficiency virus (HIV), hepatitis C virus (HCV), and hepatitis B virus (HBV). Experimental evidence suggests that insufficient signaling through the PD-1 pathway promotes immunopathology during acute infection by exaggerating primary T cell responses. If chronic infection is established, however, high levels of PD-1 expression can have unfavorable immunological consequences. Exhaustion and suppression of antiviral immune responses can result in viral immune evasion. The role of the PD-1/PD-L1 axis during viral infections is further complicated by evidence that PD-L1 also mediates inflammatory effects in the acute phase of an immune response. In this review, we discuss the intricate interplay between viruses and the PD-1/PD-L1 axis.

## Introduction

Programmed cell death 1 (PD-1, also known as CD279) was discovered by Tasuku Honjo et al. at Kyoto University from a screen of genes involved in programmed cell death (Ishida et al., 1992). PD-1 expression is rapidly induced after signaling through the T cell receptor (TCR) and modulated by cytokines (Agata et al., 1996; Yamazaki et al., 2002; Wherry et al., 2007; Chikuma et al., 2009; Terawaki et al., 2011; Ahn et al., 2018). Other types of immune cells such as B cells, natural killer (NK) cells, NKT cells, dendritic cells (DCs), and monocytes also express PD-1 (Sharpe et al., 2007; Keir et al., 2008).

There is ample evidence that PD-1, a member of the immunoglobulin superfamily, regulates the magnitude and quality of T cell responses. It plays a pivotal role in the induction and maintenance of central as well as peripheral tolerance (Nishimura et al., 1999, 2001; Wang et al., 2005; Okazaki and Honjo, 2006; Francisco et al., 2010; Fife and Pauken, 2011). For example, antigen presentation by resting DCs induces peripheral CD8+ T cell tolerance by signaling through PD-1 on CD8+ T cells (Probst et al., 2005). In fact, PD-1 has been called a ‘rheostat’ that calibrates threshold, strength, and duration of T cell responses (Okazaki et al., 2013; Honda et al., 2014). PD-1 belongs to a group of structurally different surface molecules that function as co-inhibitory receptors during immune responses against pathogens and cancer (Attanasio and Wherry, 2016; Hashimoto et al., 2018; Sharpe and Pauken, 2018). These molecules counterbalance co-stimulatory receptors on T cells such as CD28, which bind to CD80 and CD86 on professional APCs and facilitate T cell activation (Esensten et al., 2016).

Clinical studies have shown that blocking the PD-1 pathway is effective against several types of cancer including melanoma, lymphoma, lung, and renal cancer (Sanmamed and Chen, 2018). This type of treatment is referred to as immune checkpoint therapy and the blocking reagents are called immune checkpoint-inhibitors (ICIs). Together with James P. Allison, who worked on another co-inhibitory receptor called cytotoxic T-lymphocyte-associated Protein 4 (CTLA-4), Tasuku Honjo was awarded the Nobel Prize in Physiology or Medicine 2018 for the discovery of cancer therapy by inhibition of negative immune regulation (Wolchok, 2018).

PD-1 interacts with the ligands PD-L1 (CD274; also called B7-H1) and PD-L2 (CD273; also called B7-DC), which show distinct expression patterns. *In vitro*, PD-1 inhibits T cell activation by recruiting Src homology region 2-containing *protein tyrosine phosphatase* 2 (SHP2) after interaction with its ligands on APCs (Chen and Flies, 2013; Okazaki et al., 2013; Sharpe and Pauken, 2018). This is associated with dephopshorylation of crucial tyrosine residues within the CD3 complex and CD28. In virus-infected mice lacking SHP2 in T cells, however, PD-1 signaling is not impaired, suggesting the existence of redundant inhibitory pathways downstream of PD-1 (Rota et al., 2018).

PD-L1 is expressed not only by all hematopoietic cells but also by many non-hematopoietic cell types such as endothelial cells and epithelial cells (Sharpe and Pauken, 2018). In contrast, PD-L2 expression is more restricted and can be induced on hematopoietic cells such as DCs, B cells, and monocytes/macrophages. Besides PD-1, there are other known interacting partners for PD-L1 and PD-L2. PD-L1 also binds to CD80 whereas PD-L2 uses RGM domain family member B (RGMB) as an alternative binding partner. Both types of interaction also inhibit immune responses (Butte et al., 2007; Xiao et al., 2014).

Viruses have to overcome strong barriers to replicate in the hostile environment of their hosts (Virgin et al., 2009). An arsenal of weapons helps viruses to subvert antiviral immunity. This includes the exploitation of host inhibitory receptor signaling pathways (Ong et al., 2016). The impact of the PD-1/PD-L1 axis in chronic virus infections is well described whereas its role during the acute phase of viral infections is less clear (Brown et al., 2010; Attanasio and Wherry, 2016). However, whether virus-induced upregulation of PD-1 ligands represents a viral immune evasion strategy or an adaption of the host defense to minimize immunopathology is a moot point. In this review, we highlight the diverse roles of PD-1 and its ligands during virus infections and their implications for host-pathogen interaction.

## The Role of the PD-1 Pathway in Acute Virus Infections

In mice acutely infected with lymphocytic choriomeningitis virus (LCMV) strain Armstrong (LCMV Arm) PD-1 is rapidly upregulated on naïve virus-specific CD8+ T cells before they clonally expand (Ahn et al., 2018). In this model of acute LCMV infection, CD4+ T cells are not required for virus clearance, which occurs within 1–2 weeks after infection (Matloubian et al., 1994). Blockade of the PD-1 pathway at this stage further increases effector functions of CD8+ T cells by enhancing granzyme B expression and mechanistic Target of Rapamycin (mTOR) signaling. Consequently, virus elimination is accelerated although the total number of virus-specific CD8+ T cells does not change (Ahn et al., 2018). Similarly, the PD-1/PD-L axis inhibits the differentiation of CD8+ T lymphocytes into polyfunctional cytotoxic T cells during acute infection of mice with murine retrovirus (David et al., 2019). This implies that PD-1 negatively regulates the terminal differentiation of naïve CD8+ T cells into effector CD8+ T lymphocytes during acute virus infection.

After virus clearance, PD-1 expression on virus-specific T cells returns to normal levels (Barber et al., 2006; Blattman et al., 2009). The expanded pool of virus-specific effector T lymphocytes contracts due to increased cell death and memory T cells arise from a subset of fate-permissive effector T cells (Akondy et al., 2017; Omilusik and Goldrath, 2017; Youngblood et al., 2017). There are at least three major memory T cell subsets: central memory T cells (Tcm cells), effector memory T cells (Tem cells), and recently defined tissue-resident memory T cells (Trm cells). Tcm cells lack effector functions but express lymph node homing molecules and circulate through the blood and the secondary lymphoid organs (Sallusto et al., 1999). After stimulation, Tcm cells differentiate into Tem cells that lack lymph node homing molecules and continuously recirculate between blood, lymph and non-lymphoid tissues. Tem cells are bestowed with various effector functions (Sallusto et al., 1999). In contrast, Trm cells do not recirculate (Wakim et al., 2008; Gebhardt et al., 2009; Masopust et al., 2010) and express core phenotypic markers including co-inhibitory receptors such as PD-1 (Hombrink et al., 2016; Kumar et al., 2017; Pallett et al., 2017). Functionally, Trm cells participate in the first line of defense to viruses by establishing an antiviral state and recruiting circulating memory T cells to sites of viral infection (Schenkel et al., 2013, 2014; Ariotti et al., 2014; Carbone and Gebhardt, 2014). Located in multiple anatomical sites including barrier tissue such as lung, skin and gut, Trm cells are indispensable for antiviral immunity and immunosurveillance (Shin, 2018; Wu et al., 2018; Szabo et al., 2019). The functional role of the PD-1/PD-L1 axis for CD8+ Trm cells is unclear at the moment but it may prevent uncontrolled Trm activation and inflammation in virus-infected tissues and other inflammatory conditions. In accordance, blockade of PD-1 on Trm cells increases the severity of eczema in a mouse model of allergic contact dermatitis (Gamradt et al., 2019).

Intriguingly, the number of memory precursor T cells increases if PD-1 is blocked by antibodies during acute LCMV infection, possibly due to faster virus elimination (Ahn et al., 2018). Virus-specific memory CD8+ T cells that develop after the elimination of LCMV persist without antigen and are capable of self-renewal due to homeostatic proliferation in response to IL-7 and IL-15 (Wherry et al., 2004; Surh and Sprent, 2008; Abdelsamed et al., 2017). Although the blockade of the PD-1/PD-L1 axis in mice infected with LCMV Arm increases effector CD8+ T cell function, no excessive tissue damage is observed (Ahn et al., 2018). Similar to the LCMV strain WE (LCMV WE), LCMV Arm does not disseminate but instead is eliminated from infected laboratory mice after acute infection. In contrast, derivatives of LCMV Arm and LCMV WE (“clone 13” and “docile,” respectively) replicate more vigorously and persist (Matloubian et al., 1990; Welsh and Seedhom, 2008). These LCMV strains cause lethal immunopathology in mice deficient of the PD-1/PD-L1 axis (PD-L1 KO mice, PD-1 KO mice) during the acute phase of infection (Barber et al., 2006; Mueller et al., 2010; Frebel et al., 2012; Zinselmeyer et al., 2013; Shaabani et al., 2016). This is due to the killing of LCMV-infected vascular endothelium by CD8+ T cells resulting in vascular leakage with pulmonary edema and severe hypotension (Frebel et al., 2012). In a mouse model of acute viral hepatitis, the absence of PD-1 is associated not only with more rapid virus clearance but also with more severe hepatitis (Iwai et al., 2003). These results imply that the stimulation of the PD-1/PD-L1 axis during the acute phase of virus infection helps to adjust the strength and quality of the cytotoxic CD8+ T cell attack so that the good (virus elimination) and the bad (tissue damage) is balanced, preventing excessive tissue damage.

## Virus-Driven PD-L1/2 Expression

Many viruses increase PD-L1/2 expression on hematopoietic cells (Table 1) and non-hematopoietic cells (Table 2). PD-L1/2 expression is regulated by proinflammatory and anti-inflammatory signals (Sun et al., 2018). The promotor regions of PD-L1 and PD-L2, which are paralog genes, are differentially regulated although they show similarly arranged binding sites for transcription factors (Garcia-Diaz et al., 2017).

**Table 1:** Virus-induced upregulation of PD-1 ligands on hematopoietic cells.

**Virus**	**Findings**	**References**
LCMV Arm and clone13	Increased PD-L1 expression on myeloid DCs and marginal zone macrophages; decreased T cell motility in the marginal zone of the spleen due to PD-L1	Zinselmeyer et al., 2013
LCMV	High PD-L1 expression on Kupffer cells in the liver	Shaabani et al., 2016
IAV	Type I IFN induced PD-L1 expression on virus-infected professional APCs in the airways	Erickson et al., 2012; Valero-Pacheco et al., 2013; Rutigliano et al., 2014; Staples et al., 2015; McKendry et al., 2016
JEV	PD-L1 upregulation on virus-infected DCs *in vitro* and decreased expansion of Treg cells by virus-infected DCs after PD-L1 blockade	Gupta et al., 2014
EOBV	Increased numbers of PD-L1 transcripts during EOBV infection of monocytes derived from macaques	Menicucci et al., 2017
HV	PD-L1/2 upregulation on DCs; high amounts of soluble PD-1 and PD-L2 in the circulation of HV-infected patients	Raftery et al., 2018
FV	PD-L1 expression on erythroid precursor cells and CD4+ T lymphocytes	Akhmetzyanova et al., 2015
HIV	PD-L1/2 upregulation on monocytes, DCs and macrophages; Correlation between level of PD-L1 expression and disease progression	Boasso et al., 2008; Meier et al., 2008; Wang et al., 2008; Rodriguez-Garcia et al., 2011
SIV	Upregulation of PD-L1 on DCs; correlation between level of PD-L1 expression and disease progression; improved function of antiviral T cells function after PD-L1 blockade	Xu et al., 2010
HSV-1	Increased PD-L1 expression on DCs in the draining lymph nodes after virus inoculation into foot pads of mice	Channappanavar et al., 2012
VZV	PD-L1/2 upregulation on human monocytes, B cells, NK cells, and NKT cells	Jones et al., 2019
KSHV	Increased PD-L1 expression on monocytes	Host et al., 2017

*Ad, adenovirus; EOBV, Ebola virus; FV, Friend retrovirus; HIV, human immunodeficiency virus; HSV-1, herpes simplex virus type 1; HV, hantavirus; IAV, Influenza A virus; JEV, Japanese encephalitis virus; KSHV, Kaposi's sarcoma-associated herpesvirus; LCMV, lymphocytic choriomeningitis virus; LCMV Arm, LCMV strain Armstrong; LCMV clone13, LCMV strain clone13; RSV, respiratory syncytial virus; Treg cells, regulatory T cells; VZV, varicella zoster virus*.

**Table 2:** Virus-induced PD-L1 upregulation on non-hematopoietic cells.

**Viruses**	**Findings**	**References**
LCMV	PD-L1 upregulation on fibroblastic reticular cells	Zinselmeyer et al., 2013
Ad	Increased PD-L1 expression on primary human hepatocytes	Grakoui et al., 2006; Muhlbauer et al., 2006
HBV	Upregulated PD-L1 expression on hepatocytes derived from a transgenic mouse model of BV infection	Maier et al., 2007
IAV, MHPV, PIV-3, RSV	Increased levels of PD-L1 on alveolar and bronchiolar epithelial cells after virus infection *in vitro* and in patients with viral acute lower tract infections	Stanciu et al., 2006; Telcian et al., 2011; Erickson et al., 2012; McNally et al., 2013
RABV	Type I IFN-dependent PD-L1 upregulation on virus-infected mouse and human neuronal cells *in vitro* and on neuronal cells in virus-infected mice	Lafon et al., 2008
HSV-1	PD-L1 upregulation on mouse neuroblastoma cells	Chentoufi et al., 2011
HSV-1	PD-L1 upregulation on virus-infected neurons in ganglia	Jeon et al., 2013
HSV-1	PD-L1 upregulation on epithelial cells in the virus-infected cornea	Jeon et al., 2018

*Ad, adenovirus; EOBV, Ebola virus; HMPV, human metapneumovirus; HBV, hepatitis B virus; HSV-1, herpes simplex virus type 1; HV, hantavirus; IAV, Influenza A virus; JEV, Japanese encephalitis virus; LCMV, lymphocytic choriomeningitis virus; LCMV Arm, LCMV strain Armstrong; LCMV WE, LCMV strain WE; PIV-3, parainfluenza virus type 3; RABV, rabies virus; RSV, respiratory syncytial virus; Treg cells, regulatory T cells; VHF, viral hemorrhagic fever*.

Type I and type III interferons (IFNs) are important antiviral cytokines. They are induced early in virus-infected barrier tissue such as lung/gut epithelial cells and serve as the first line of antiviral defense (Okabayashi et al., 2011; Wack et al., 2015; Andreakos et al., 2017; Galani et al., 2017; Zanoni et al., 2017; Good et al., 2019; Lazear et al., 2019). Type I IFNs, which in humans include several IFN-α subtypes and IFN-β, increase PD-L1 expression but to a lesser extent than PD-L2 expression (Garcia-Diaz et al., 2017). PD-L2 responds equally well to IFN-γ (type II IFN) and IFN-β (Garcia-Diaz et al., 2017). IL-4 may be an even more potent inducer of PD-L2 (Loke and Allison, 2003) thus accounting for the presence of PD-L2 on monocyte-derived DCs generated *in vitro*. Blockade or absence of type I IFN signaling during chronic LCMV infection results in reduced PD-L1 expression despite enhanced viral replication (Teijaro et al., 2013; Wilson et al., 2013; Shaabani et al., 2016). Although type I IFNs moderately upregulate PD-L1 (Sun et al., 2018) they increase NK cytotoxicity and allow clonal expansion and memory formation of antiviral cytotoxic CD8+ T cells (Biron et al., 2002; Kolumam et al., 2005; Aichele et al., 2006). Type III IFNs signal through a unique heterodimeric receptor and induce the expression of antiviral IFN*-*stimulated genes *(*ISGs) similar to type I IFNs (Davidson et al., 2015). Intriguingly, type III IFNs do not upregulate PD-L1 (Raftery et al., 2018). Accordingly, in this early phase of acute infection the PD-1/PD-L1 axis does not inhibit antiviral immune cells.

Recognition of viruses by pattern recognition receptors (PRRs) also upregulates PD-L1. TLR3 signaling in particular strongly increases PD-L1 levels on DCs (Pulko et al., 2009; Boes and Meyer-Wentrup, 2015; Raftery et al., 2018) whereas RIG-I signaling alone has no significant effect (Raftery et al., 2018). Triggering of TLR3, which transmits downstream signals through the TIR-domain-containing adapter-inducing IFN-β (TRIF), also enhances PD-L1 on other cell types including endothelial cells (Cole et al., 2011) and epithelial cells (Tsuda et al., 2005). In accordance, virus-induced PD-L1 upregulation on neuronal cells is severely impaired in TLR3-deficient mice (Lafon et al., 2008). Recently, viral proteins inducing PD-L1/PD-L2 expression have been identified. For example, HIV Tat protein increases PD-L1 expression on DCs through TNF-α and TLR4 signaling (Planes et al., 2014). The HCV core protein *in vitro* induces strong PD-L1 upregulation on primary human Kupffer cells and monocytes in a TLR2- and PI3K-dependent manner (Tu et al., 2010; Zhai et al., 2017). In accordance, the PD-L1 levels on monocytes from HCV-infected patients were significantly higher than on monocytes from healthy individuals (Zhai et al., 2017). A recent study has shown that extracellular vesicles (EVs) produced by HBV-infected hepatocytes are endocytosed by circulating monocytes resulting in PD-L1 upregulation (Huang et al., 2017; Kakizaki et al., 2018). Moreover, PD-L1 and PD-L2 are upregulated by hantaviral N protein most likely via hantavirus-induced TLR3 signaling (Raftery et al., 2018). In addition, the latency-associated transcripts (LATs) of herpes simplex virus type 1 (HSV-1) upregulate PD-L1 on mouse neuroblastoma cells by an unknown mechanism (Chentoufi et al., 2011). Remarkably, as of yet no viral immunoevasin has been discovered that directly interacts with the molecules of the PD-1/PD-L1 axis to exploit its immunosuppressive function.

Viral replication can also result in the production of anti-inflammatory cytokines such as IL-10 (Brooks et al., 2006b; Ejrnaes et al., 2006). Cellular IL-10 has been shown to upregulate the expression of PD-1 and PD-L1 in a STAT-3 dependent manner in DCs and monocytes (Curiel et al., 2003; Selenko-Gebauer et al., 2003; Sun et al., 2015; Lamichhane et al., 2017). Accordingly, the absence of cellular IL-10 in LCMV infected mice results in enhanced effector T cell responses, rapid virus elimination, and generation of antiviral memory T cells (Brooks et al., 2006b; Ejrnaes et al., 2006). Intriguingly, during coevolution with their hosts members of the virus family *Herpesviridae* have acquired numerous genes from their hosts including those that mimic cellular IL-10 (Raftery et al., 2000; Ouyang et al., 2014; Schonrich et al., 2017). These viral IL-10 (vIL-10) molecules act as immunosuppressive cytokines that also paralyze co-stimulatory B7 molecules (Muller et al., 1998; Raftery et al., 2004). It is possible that vIL-10 molecules also increase signaling through the PD-1/PD-L1 axis similar to their cellular counterparts thereby contributing to viral persistence. However, combined blockade of both, IL-10 and PD-L1, during chronic LCMV infection enhances T-cell function more efficiently than a single blockade (Brooks et al., 2008). Thus, IL-10 is pleiotropic and has immunosuppressive functions independent of the PD-1/PD-L1 axis during persisting virus infections (Ouyang et al., 2011).

In the late phase of acute virus infection, type II IFN and several other cytokines including TNF-α and IL-10 are released by immune cells such as CD8+ T cell cells (Zhang and Bevan, 2011). IFN-γ strongly upregulates PD-L1 (Garcia-Diaz et al., 2017; Raftery et al., 2018; Sun et al., 2018). In addition, plasmacytoid DCs (pDCs) migrate into virus-infected tissue and secrete large amounts of type I IFNs (Siegal et al., 1999). These cytokines not only induce antiviral ISGs but also drive inflammatory responses such as secretion of TNF-α, IL-1β, or IL-6 (Davidson et al., 2015), which can further increase PD-L1 on various cell types including endothelial cells and at the same time promote non-lytic virus elimination (Sun et al., 2018). Thus, in the late phase of acute viral infection, PD-L1 is strongly upregulated thereby downregulating terminal differentiation of CD8+ T cells and preventing excessive tissue damage due to uncontrolled cytotoxic attack.

## Function of PD-L1 During Acute Virus Infections

PD-L1 expressed on hematopoietic or non-hematopoietic cells has different functions (Keir et al., 2006; Mueller et al., 2010). For example, PD-L1 expression on parenchymal cells of the pancreas rather than hematopoietic cells prevent autoimmune diabetes (Keir et al., 2006). In accordance, during LCMV infection of mice PD-L1 expression on non-hematopoietic cells reduces viral clearance and immunopathology (Keir et al., 2008). Thus, upregulation of PD-L1 expression may protect virus-infected cells from being eliminated by cytotoxic CD8+ T cells. On the other hand, selective absence of PD-L1 on hematopoietic cells results in lethal immunopathology (Mueller et al., 2010). This is best explained by an increase in number and function of cytotoxic CD8+ T lymphocytes, which may overwhelm the PD-L1-conferred protection in non-hematopoietic target cells (Frebel et al., 2012).

**Figure 1:**
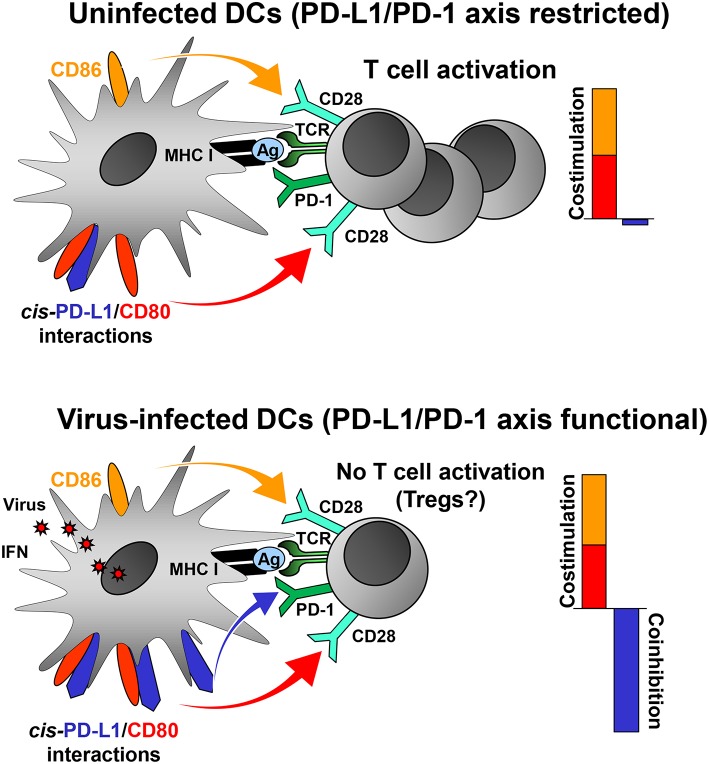
PD-L1 mediated viral regulation of T cell activation. Upper graph: In the absence of viral infection mature dendritic cells (DCs) express relatively low levels of PD-L1. Recognition of cognate antigen (Ag) bound to MHC class I molecules by T cell receptor (TCR) results in upregulation of PD-1 on T cells. DCs express co-stimulatory molecules CD80 and CD86 allowing efficient co-stimulation of T cells via CD28. The PD-1/PD-L1 axis is not co-inhibitory due to restriction by *cis*-PD-L1/CD80 interactions, and thus T cells are activated. Lower graph: In the context of viral infection DCs upregulate PD-L1 due to exposure to viral PAMPs and high levels of type I IFN. The restricting *cis*-PD-L1/CD80 interactions are most likely overwhelmed by virus-induced PD-L1 resulting in PD-1 signaling and prevention of T cell activation. The consequences of this for the generation of Tregs is as of yet unknown.

Virus-induced PD-L1 on professional APCs may help to focus the antiviral CD8+ T cell response on a few strongly stimulatory, i.e., immunodominant, virus-derived epitopes by increasing the threshold of CD8+ T cell activation. In this way, the majority of weakly immunogenic viral peptides fail to activate CD8+ T cells. The adjustment of the “rheostat” on professional APCs may be necessary to prevent autoimmune disease and maintain peripheral tolerance in the face of a highly inflammatory milieu. Indeed, a recent study has shown that the PD-L1/PD-1 axis regulates T cell responses at the activation stage (Sugiura et al., 2019). CD80, which binds to CD28 and CTLA-4 on T cells, also interacts with PD-L1 (Butte et al., 2007, 2008). Importantly, this interaction occurs only in *cis* (Chaudhri et al., 2018) and prevents PD-L1 on DCs from co-inhibitory signaling to T cells via PD-1 (Chaudhri et al., 2018; Sugiura et al., 2019). In contrast, the functions of CD28 (co-stimulatory) and CTLA-4 (co-inhibitory) are not impaired by *cis*-PD-L1/CD80 interactions on DCs (Sugiura et al., 2019). Many viruses upregulate PD-L1 on professional APCs such as DCs (Table 1) either directly or through IFN release. Low PD-L1 levels on uninfected DCs have only a weak impact on T cell activation (Brown et al., 2003) due to *cis*-PD-L1/CD80 interactions (Figure 1, upper scheme). It is likely, that the high PD-L1 levels on DCs in the context of viral infection will overwhelm the *cis*-binding capacity of CD80 resulting in increased co-inhibitory signaling via PD-1 (Figure 1, lower scheme). PD-L1 on professional APCs also promotes the induction and maintenance of regulatory T cells (Treg cells; Francisco et al., 2009). Treg cells help to confine the antiviral defense and to prevent immunopathology during virus infections (Veiga-Parga et al., 2013). Taken together, viruses can reprogram DC function in antiviral immune responses by tipping the balance between co-inhibitory and co-stimulatory signals as shown for murine cytomegalovirus (Loewendorf et al., 2004; Benedict et al., 2008) and vaccinia virus (Kleinpeter et al., 2019).

Strong stimulation of the PD-1/PD-L1 does not prevent immunopathology during viral hemorrhagic fever (VHF). VHF is a designation for distinct but pathogenically similar zoonotic diseases that are caused by several enveloped RNA viruses including Ebola virus (EBOV), hantavirus, and dengue virus (DENV) (Paessler and Walker, 2013). VHF viruses target endothelial cells thereby causing vascular leakage (Zampieri et al., 2007; Schonrich et al., 2008; Basler, 2017). In fact, type III IFN and TNF-α, which upregulate PD-L1 on endothelial cells, also mediate dysfunction of the endothelial barrier (Brett et al., 1989; Koh et al., 2004). Virus-specific CD8+ T cells show high levels of PD-1 on the surface during acute infection with EBOV (McElroy et al., 2015). Moreover, fatal EBOV infection is characterized by a high percentage of T cells expressing PD-1 and other co-inhibitory receptors such as CTLA-4 (Ruibal et al., 2016). Monocytes are susceptible to EBOV infection and upregulate production of PD-L1 transcripts in response to EBOV replication (Menicucci et al., 2017), whereas DENV-infected DCs express higher levels of PD-L2 but reduced PD-L1 (Nightingale et al., 2008). In patients with acute hantavirus infection large amounts of soluble PD-L1/PD-L2 are found in the sera indicating that these molecules are strongly upregulated in hantavirus-infected cells *in vivo* (Raftery et al., 2018). In accordance, strongly increased PD-L1 levels are detected after hantavirus infection of immature DCs *in vitro* and in hantavirus-infected mice with a humanized immune system (Raftery et al., 2018). In striking contrast, CD8+ T cells do not upregulate PD-1 during acute hantavirus infection (Lindgren et al., 2011).

**Figure 2:**
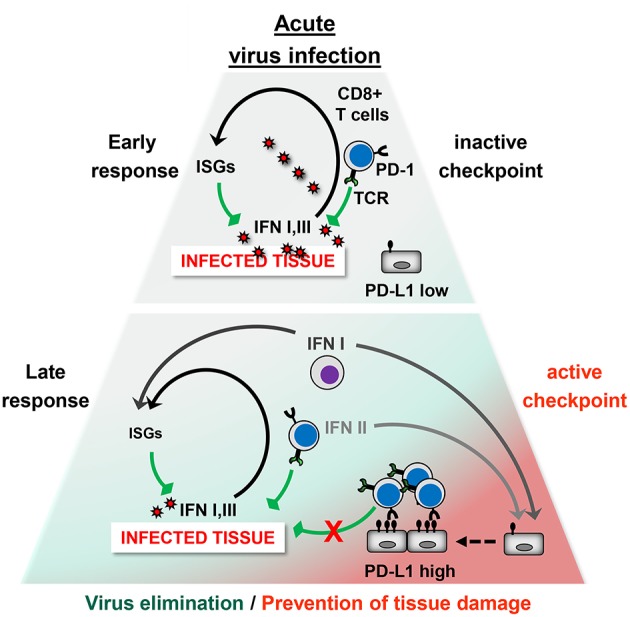
The PD-1/PD-L1 checkpoint in acute virus infection. Early phase: The infected tissue produces type I IFNs and possibly type III IFNs, which strongly induce antiviral IFN*-*stimulated genes (ISGs) but only moderate PD-L1 levels. Antiviral CD8+ T cells eliminate virus-infected cells. At this stage, the PD-1/PD-L1 checkpoint activity is low and does not restrict the antiviral immune response. Late Phase: Type II IFN and TNF-α is secreted by CD8+ T cells and other immune cells. In addition, hematopoietic cells such as plasmacytoid DCs (pDCs) produce large amounts of type I IFN. This results not only in virus elimination but also increases PD-L1 expression. The high checkpoint activity downregulates terminal differentiation of antiviral CD8+ T cells. Ideally, the strength and quality of the CD8+ T cell response is balanced out in such a way that the viral intruder is eliminated without causing immunopathology.

Taken together, in the acute phase of viral infection virus-specific T cells rapidly upregulate the co-inhibitory receptor PD-1 upon recognition of antigen. Simultaneously, viruses upregulate PD-L1 on hematopoietic and non-hematopoietic cells directly through PRR signaling or indirectly by inducing the release of IFNs and other inflammatory cytokines. Ideally, a tailor-made antiviral CD8+ T cell response eliminates viral pathogens with minimal immunopathology (Figure 2). The antiviral immune response during VHF, however, eliminates viruses at the cost of vascular leakage. The dysregulation of the immune responses could be due to variations in PD-L1 expression (e.g., timing, cell type, or strength), imbalance between co-stimulatory vs. co-inhibitory receptors (failure of “checks and balances”), or altered usage of PD-L1 interaction partners (PD-1, CD80, and possibly additional unknown partners). On the other hand viruses can also manipulate the “checks and balances” of the immune system in such a way that an effective antiviral immune response is prevented helping the pathogen to persist in the organism.

## The PD-1/PD-L1 Axis During Persisting Virus Infections

### Chronic Virus Infection

Chronic infections with viruses such as hepatitis B virus, hepatitis C virus (HCV), and human immunodeficiency virus (HIV) represent major causes of chronic disease and death worldwide (Ott et al., 2012; Schweitzer et al., 2015; GBD 2015 HIV Collaborators, 2016; Stanaway et al., 2016). During chronic infection virus particles are continuously released from virus-infected cells and maintain a network of immunosuppressive mechanisms that interfere with virus elimination (Ng et al., 2013). Therefore, T cells enter a state called T cell exhaustion.

### T Cell Exhaustion and Partial Restoration of T Cell Function by Blockade of the PD-1/PD-L1 Axis

The first evidence for T cell exhaustion was gathered in paradigmatic experiments using LCMV-infected mice (Zehn and Wherry, 2015; Kahan and Zajac, 2019). Derivatives of LCMV Arm and LCMV WE (LCMV clone13 and LCMV docile, respectively) vigorously replicate and disseminate in mice thereby persisting for more than 100 days (Moskophidis et al., 1993; Gallimore et al., 1998; Zajac et al., 1998). In this model of chronic virus infection, CD4+ T cells are crucial to sustain the virus-specific CD8+ T cell responses (Matloubian et al., 1994). Sustained upregulation of PD-1 and other co-inhibitory receptors such as 2B4, CTLA-4, and lymphocyte-activation gene 3 (Lag3) has become the defining characteristic of exhausted T (Tex) cells (Barber et al., 2006; Wherry et al., 2007; Blackburn et al., 2009; Crawford et al., 2014). These phenotypic changes are accompanied by a multistep loss of T cell effector functions (Speiser et al., 2014; Kahan et al., 2015; Wherry and Kurachi, 2015; McKinney and Smith, 2018). Dependent on the strength of PD-1 signaling CD8+ T lymphocytes gradually lose important effector functions (Wherry et al., 2003; Wei et al., 2013). Some are lost early (such as cytotoxicity, IL-2 production, and proliferation), whereas others (e.g., IFN-γ production) are maintained for a longer time (Wherry et al., 2003; Wei et al., 2013). Finally, Tex cells undergo apoptosis (Kahan et al., 2015). As an underlying mechanism of T cell exhaustion during chronic LCMV infection, PD-1 signaling impairs T cell motility facilitating engagement of inhibitory pathways in T cells (Zinselmeyer et al., 2013). In another experimental setting, PD-L1 blocking antibodies prolong the T cell migration arrest suggesting that PD-1 signaling in fact enhances T cell motility (Honda et al., 2014). The reason for these contrasting results are unclear at the moment. Intriguingly, PD-1-regulated changes in several metabolic pathways occur at the very beginning of Tex cell development underlining the importance of these metabolic processes in the execution of the Tex program (Bengsch et al., 2016; Schurich et al., 2016;McKinney and Smith, 2018).

Several reports have found differences in the transcriptional program and epigenetic profile of Tex cells as compared to memory and effector T lymphocytes (Wherry et al., 2007; Doering et al., 2012; Pauken et al., 2016). In Tex cells derived from LCMV-infected mice, the *Pdcd1* regulatory region is completely demethylated and remains so even when virus titers decrease (Youngblood et al., 2011). They do not show antigen-independent persistence driven by IL-7 and IL-15, the hallmark of memory T cells, and instead require the continuous presence of their cognate antigen (Wherry and Ahmed, 2004; Shin et al., 2007). This can be explained by the observation that the TCR-induced transcription factors IRF4, BATF, and NFATc1 not only drive T cell exhaustion but also impair memory T cell development during chronic LCMV infection (Man et al., 2017). Recently, microRNA (miR)-155 has been identified as a key molecule that promotes long-term persistence of Tex cells (Stelekati et al., 2018).

T cells that have been rendered dysfunctional during persisting virus infections can be reinvigorated (Brooks et al., 2006a). Blockade of the PD-1/PD-L1 axis during chronic LCMV infection reinvigorates antiviral T cell functions and reduces viral load (Barber et al., 2006). Of note, CD8+ T cells also become exhausted in the absence of PD-1 (Odorizzi et al., 2015). These experiments show that other coinhibitory receptors contribute to T cell exhaustion. In line with this view, a combined blockade of PD-1 and LAG-3 or PD-1 and Tim-3 synergistically improves antiviral CD8+ T cell responses and viral control in mice with chronic LCMV infection (Blackburn et al., 2009; Jin et al., 2010). Reinvigorated CD8+ T cells in chronically LCMV-infected mice become exhausted again after termination of the PD-L1 blockade (Pauken et al., 2016; Sen et al., 2016; Turner and Russ, 2016). This finding indicates that inflexibility of the epigenetic regulation in Tex cells may limit the success of therapies using ICIs.

The studies of chronic LCMV infection in mice also relate to important human infectious diseases. In a recently established mouse model of HCV infection Tex cells are observed in the liver of mice infected with a newly identified Norway rat hepacivirus (NrHV), which belong to the same virus family as HCV (Billerbeck et al., 2017; Klenerman and Barnes, 2017). In NrHV-infected mice, CD4+ T cells were important to maintain the antiviral CD8+ T cell response similar to the LCMV model of chronic virus infection (Billerbeck et al., 2017). Blockade of the PD-1/PD-L1 axis in early chronic infection reduced the viral load whereas no beneficial effects were observed at later time points (Billerbeck et al., 2017). Moreover, ICIs blocking the PD-1 pathway can reinvigorate to some extent Tex cells in humans chronically infected with HBV or HCV (McKinney and Smith, 2016; Cox et al., 2017; Saeidi et al., 2018; Wykes and Lewin, 2018). Targeting the PD-1/PD-L1 pathway during retroviral infections has beneficial effects for virus control (Velu et al., 2015). PD-1 upregulation is linked to a loss of function in HIV-specific CD8+ T cells, which can be partially reversed *in vitro* by a blockade of the PD-1/PD-L1 axis (Day et al., 2006; Trautmann et al., 2006). Surprisingly, the context and timing of PD-1 blockade seems to be important for its functional outcome: PD-1 signaling inhibition during stimulation of naive CD8^+^ T cells results in diminished activation, whereas PD-1 blockade during the T cell effector phase increases activation (Garcia-Bates et al., 2019). PD-1 blockade in rhesus macaques infected with simian immunodeficiency (SIV) rapidly increases the number and functional quality of virus-specific CD8+ T cells (Velu et al., 2009). Intriguingly, the combination of anti-PD-1 antibodies and antiretroviral therapy further improves antiviral CD8+ T cell function in SIV-infected rhesus macaques (Mylvaganam et al., 2018). This observation implies that directly acting antivirals (DAAs) reducing the viral load and ICIs releasing the brake in Tex cells synergistically increase antiviral T cell responses.

Recent data suggest that PD-1 expression does not necessarily reflect T cell failure but rather adaption of T cell function to chronic inflammation (Utzschneider et al., 2013, 2016; Speiser et al., 2014; Staron et al., 2014; Zehn et al., 2016; Barnes, 2018; Petrelli et al., 2018). In fact, at least two CD8+ Tex cell subsets exist that act in concert to mount a partially effective CD8+ T cell response for control of chronic virus infection (Paley et al., 2012). Moreover, Tex cells have the capacity for self-renewal and are not entirely functionally inactive (Paley et al., 2012). The latter finding implies that Tex cells may represent a form of antiviral defense that is evolutionary adapted to the need to control a chronically replicating non-lytic virus with minimal collateral tissue damage and immunopathology. Moreover, experiments in mice with genetic ablation of PD-1 suggest that PD-1 is not required for induction of Tex cells (Odorizzi et al., 2015). In fact, PD-1 may play a pivotal role in maintaining Tex cells by preventing excessive stimulation that leads to proliferation and terminal differentiation (Odorizzi et al., 2015). After the elimination of HCV by DAAs, PD-1 expressing CD8+ T cell populations remain that display characteristics of memory cells including antigen-independent survival and proliferation after re-challenge with antigen (Wieland et al., 2017).

### Latent Infection and Reactivation

Viruses that establish latent infection include the members of the family *Herpesviridae*. In contrast to chronic infection, latent infection is characterized by periodic suspension of virus replication. However, the blueprint of viral particles is preserved in the latently infected host cells enabling the virus to reactivate and resume virus production. It is a matter of debate whether reactivation from latent virus infection creates enough antigenic load to induce exhaustion of antiviral CD8+ T cells. Memory CD8+ T cells recognizing viral antigens in the context of chronic virus infections (e.g., HIV) more frequently express PD-1 than memory CD8+ T cells stimulated by virus periodically reactivating from latency, e.g., human cytomegalovirus (HCMV) (Petrovas et al., 2006). This finding is consistent with the idea that the amount of available antigen regulates PD-1 expression on reactive T cells (Petrovas et al., 2006). In accordance, increased virus replication in immunosuppressed patients with HCMV disease after allogeneic hematopoietic cell transplantation is associated with PD-1 upregulation on T cells (Gallez-Hawkins et al., 2009). In mice with a humanized immune system, HCMV reactivations induced by granulocyte-colony stimulating factor (G-CSF) resulted in a shift toward PD-1 expressing T cells (Theobald et al., 2018). Whether this phenotype corresponds to Tex cells is unclear, however. Upregulation of co-inhibitory receptors such as PD-1 on CD8+ T cells is tightly linked to activation and differentiation and not *per se* proof of T cell exhaustion (Legat et al., 2013). In addition, studies of HSV-1 infection in mice did not reveal evidence for functional impairment of virus-specific CD8+ T cells during latency and subsequent reactivations (Mackay et al., 2012).

PD-1 expression on brain Trm cells is maintained independently from antigen (Shwetank et al., 2017). Recently, it has been shown that Trm cells provide immunosurveillance in the human brain to eliminate neurotropic viruses (Smolders et al., 2018). In accordance, reactivation of HSV-1 from latently infected neurons of the mouse is controlled by CD8+ Trm lymphocytes (Liu et al., 2000; Khanna et al., 2003; Verjans et al., 2007). These immune cells provide IFN-γ which upregulates PD-L1 on HSV-1-infected neurons (Jeon et al., 2013). CD8+ T cells recognizing subdominant epitopes derived from HSV-1 proteins other than glycoprotein B (gB) but not CD8+ T cells specific for the dominant gB-derived epitope show a partial exhausted phenotype with increased PD-1 expression (Jeon et al., 2013). Blockade of PD-L1 resulted in increased survival of exhausted CD8+ T cells that were non-functional and not protective, however (Jeon et al., 2013). In contrast, it has been reported that HSV-1 LATs promotes functional exhaustion of CD8+ T cells specific for the dominant gB-derived epitope (Allen et al., 2011; Chentoufi et al., 2011).

During coevolution with their host, herpesviruses developed numerous mechanisms to evade the antiviral immune response such as modulation of programmed cell death (Raftery et al., 1999, 2001; Muller et al., 2004; Kather et al., 2010) and downregulation of MHC class I molecules (Schuren et al., 2016). Intriguingly, replication competent varicella-zoster virus (VZV) downregulates MHC class I and PD-L1 molecules in human brain vascular adventitial fibroblasts, perineurial cells, and human lung fibroblasts (Jones et al., 2016). In contrast, VZV upregulates PD-L1 in hematopoietic cells (Jones et al., 2019). The mechanism underlying VZV-associated downregulation of PD-L1 is posttranscriptional in nature but the VZV-encoded protein responsible has not yet been identified (Jones et al., 2016). VZV might target PD-L1 to increase the migration arrest of T cells (Honda et al., 2014). In this way, the virus could more efficiently spread from lung fibroblasts to T cells, which play crucial role in VZV dissemination to the skin (Arvin et al., 2010).

## Concluding Remarks

It is a seductive proposition that a virus induces PD-1 ligands in order to inhibit and thus evade the host immune response. On the other hand, recent data on the regulation of PD-L1 expression during viral infection suggest that PD-L1 upregulation is rather a part of the normal innate response induced by IFNs and PRR signaling. The reason for this is still enigmatic. PD-L1 may have a yet not defined immunostimulatory role in the very early phase of viral infection. Later, it may adjust the quantity and quality of the antiviral CD8+ T cell response in such a way that virus is eliminated with minimal collateral tissue damage. The PD-1/PD-L1 axis may also be important to maintain antiviral Trm cells and Tex cells. Virus-induced PD-1 ligand expression as an immune evasion strategy should always be rigorously tested with this in mind.

## Author Contributions

All authors listed have made a substantial, direct and intellectual contribution to the work, and approved it for publication.

### Conflict of Interest Statement

The authors declare that the research was conducted in the absence of any commercial or financial relationships that could be construed as a potential conflict of interest.
